# Copy number variations play important roles in heredity of common diseases: a novel method to calculate heritability of a polymorphism

**DOI:** 10.1038/srep17156

**Published:** 2015-11-24

**Authors:** Yoshiro Nagao

**Affiliations:** 1 Department of Pediatrics, Takashimadaira Chuo General Hospital, 1-73-1 Takashimadaira, Itabashi, Tokyo 1750082, Japan; 2 Department of Pediatrics, The University of Tokyo, Bunkyo, Tokyo, Japan; 3 Department of Clinical Genetics, Tokai University, Isehara, Kanagawa, Japan

## Abstract

“Missing heritability” in genome wide association studies, the failure to account for a considerable fraction of heritability by the variants detected, is a current puzzle in human genetics. For solving this puzzle the involvement of genetic variants like rare single nucleotide polymorphisms (SNPs) and copy number variations (CNVs) has been proposed. Many papers have published estimating the heritability of sets of polymorphisms, however, there has been no paper discussing the estimation of a heritability of a single polymorphism. Here I show a simple but rational method to calculate heritability of an individual polymorphism, h_p_^2^. Using this method, I carried out a trial calculation of h_p_^2^ of CNVs and SNPs using published data. It turned out that h_p_^2^ of some CNVs is quite large. Noteworthy examples were that about 25% of the heritability of type 2 diabetes mellitus and about 15% of the heritability of schizophrenia could be accounted for by one CNV and by four CNVs, respectively. The results suggest that a large part of missing heritability could be accounted for by re-evaluating the CNVs which have been already found and by searching novel CNVs with large h_p_^2^.

Genome-wide association studies (GWAS) have identified hundreds of gene polymorphisms associated with common diseases, however, every effort to explain the heritability of a disease by single nucleotide polymorphisms (SNPs) detected in GWAS has been failed^1^,^2^,^3^. Wellcome Trust Case Control Consortium *et al.* reported a genome-wide association study of copy number variations (CNVs) for eight common diseases in 2010, and they concluded that common CNVs that can be typed on existing platforms are unlikely to contribute greatly to the genetic basis of common human diseases^4^. Because efforts have largely focused on common genetic variants, one hypothesis is raised that much of the missing heritability is due to rare genetic variants^2^,^5^. However, it has not yet reported that a large part of the heritability of a disease is accounted for by rare variants. Although many papers have reported the contribution of a set of variants to heritability by the quantitative genetic analysis, there has been no paper discussing about the estimation of a heritability of a single polymorphism. Here I describe a novel method to calculate heritability of an individual polymorphism including a SNP or a CNV.

## Results

### Definitions and premises

- The frequency of a risk allele in a general population: p.
- The frequency of non-risk allele in a general population: q.
- The frequency of a risk allele in patients: u.
- The frequency of non-risk allele in patients: v.
- The prevalence of a disease: **P**. Suppose frequencies of the risk and non-risk alleles of asymptomatic individuals are represented by x and y, respectively, then the following relationships are generated:

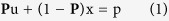



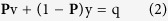


Odds ratio, OR, is represented by the following:


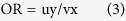


In the reports of case-control study, u, x, and OR are usually shown, and p can be calculated by using Equation [1]. When the data of p and OR are available in a SNP database, u or v should be calculated. It is impossible to have reasonable solutions of u and v using Equations [1, 2, 3]. Instead, they can be estimated by approximated solutions. First of all, calculation of genotype frequencies of the first-degree relatives is necessary for the estimation of heritability. For this purpose, Bayes’ method will be needed, because frequency of the risk genotype(s) of them should be calculated with a posterior probability. For these purposes the following definitions are needed.

- A and a represent dominant and recessive allele, respectively.
- The genotype frequency of AA for the proband: α.
- The genotype frequency of Aa for the proband : β.
- The genotype frequency of aa for the proband: γ.
- The frequency of the risk genotype(s) of the general population: X_1_.
- The frequency of the risk genotype(s) of the first-degree relatives: Y_1_.

The probability of each genotype for a sibling and an offspring is shown in Table 1. The probability of each genotype for a parent, that is same as for an offspring, is omitted here. The calculation procedure to have genotype probabilities were shown in the section of the methods.

Then the calculations of the heritability of a polymorphism of the main subject are shown.

### Heritability of a polymorphism under an autosomal dominant (AD) model

When genotypes AA and Aa have a same risk effect, Y_1_ of a sibling is calculated using the expressions in Table 1 as follows:


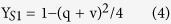


Y_1_ of an offspring is calculated as follows:


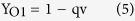


A relation between the arithmetic mean and the geometrical average indicates that there is a relation of Y_O1_ > Y_S1_ unless v equals to q.

**Figure 1:**
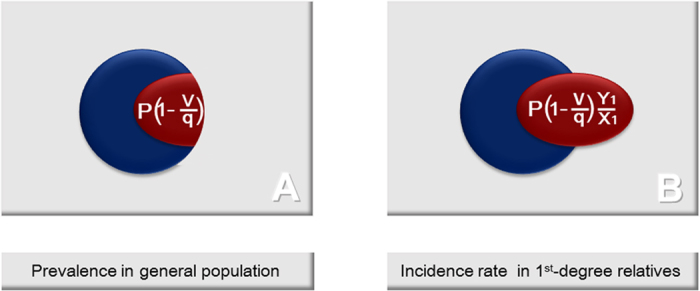
Schematic images of the prevalence of a disease in general population, P, and the incidence rate of the disease for first-degree relatives, Q, for a polymorphism. **(A) P** is represented by a circle. The area where the population attributable risk of a polymorphism, **P**(1–v/q), covers is applied gray. **(B) Q** is represented by the area where either the circle or the gray oval covers. **Q** is bigger than **P** by **P**(1−v/q)(Y_1_/X_1_−1). **q**: allele frequency of the non-risk allele for the general population. **v**: allele frequency of the non-risk allele for the patient group. **X**_**1**_: frequency of the risk genotype of the general population. **Y**_**1**_: frequency of the risk genotype of the first-degree relatives.

Let us think about the incidence rate of the disease among the first-degree relatives, **Q**. When a polymorphism is involved in a part of the patients group, its share in the prevalence, **P**, is represented by the population attributable risk that is denoted by **P**(1–v/q) (Fig. 1A). Suppose that the risk allele of a polymorphism is the only genetic cause of a disease. For the first-degree relatives of the patients who do not have the risk allele the incident rate is not different from that in the general population. Therefore **Q** will be bigger than **P** by (Y_1_/X_1_ − 1) for the effect of this polymorphism (Fig. 1B). Then the incidence rate of the disease for a sibling, **Q**_**s**_, is represented by Equation [6], as follows:


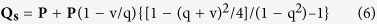


The incidence rate for an offspring, **Q**_**o**_, is represented by Equation [7], as follows:


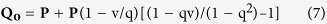


Once **Q**_**s**_ or **Q**_**o**_ is estimated, the heritability of a polymorphism, h_p_^2^, is calculated by the Falconer’s liability threshold model^6^.

### Heritability of a polymorphism under an autosomal recessive (AR) model

It is known that some polymorphisms show a recessive effect. If the risk allele of a polymorphism shows a recessive effect, frequencies of the risk genotypes of a sibling and an offspring, Y_S1_ and Y_O1_, are represented as follows, respectively:


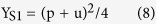



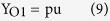


In the recessive model, homozygote is the risk genotype. Therefore the proportion of patients who have the risk genotype in the holder of risk allele is represented by u^2^/(u^2^ + 2uv). The incidence rates of the disease among siblings and among offspring, if we consider only for the effect of the polymorphism are represented by next Equations, respectively, as follows:


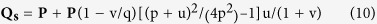



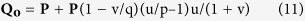


### Heritability of a polymorphism under other inheritance models

h_p_^2^ can be estimated for a polymorphism under any other inheritance models so far the frequency of the risk genotype(s) for the first-degree relatives can be calculated. If a polymorphism is located on an autosome and if the OR of heterozygote is smaller than that of homozygote, the h_p_^2^ of this polymorphism is smaller than h_p_^2^ under AD model and larger than h_p_^2^ under AR model.

### Calculation of the heritability of two or more polymorphisms

Falconer’s method is based on the calculation of the “liability thresholds” for the prevalence of a disease in general population and for the recurrence rate in the first-degree relatives. Units of these measures are standard deviations and heritability is estimated by the difference of two measures^6^. The calculation of the heritability of two or more polymorphisms is possible. For this purpose second clause of Equation [6] or [7] for each polymorphism should be calculated and added finally to **P.**

### Estimation of various CNVs and SNPs reported in the literatures

Most germline CNVs are heritable^7^. However, heredity form of a CNV is not always known. Furthermore a *de novo* CNV is sometimes identified in the association studies (3). The heritability of a disease has been often estimated by twin studies. Monozygotic (MZ) twins share all germline polymorphisms including *de novo* variants, whereas dizygotic (DZ) twins usually do not share a *de novo* polymorphism. Because heritability is calculated by a difference between the concordance rates of MZ twins and DZ twins, a *de novo* polymorphism should also be involved in the estimation of heritability in a twin study. When we estimate the contribution of a CNV to the heritability of a disease by Falconer’s model, the recurrence risk to hold the CNV for a sibling cannot be used theoretically because it may be a *de novo* CNV for the proband. On the other hand, the recurrence risk for an offspring can be used because all germline polymorphisms, including *de novo* ones, will be fundamentally transmitted to the offspring.

Table 2 listed various CNVs and SNPs reported in the literatures. The h_p_^2^ of these polymorphisms were calculated for offspring under the AD model. As shown in Table 2, CNVs generally have a larger h_p_^2^ (>0.01). A noteworthy result was that about 25% of the heritability of type 2 diabetes mellitus (T2DM) could be accounted for by one CNV, a value greater than the previously estimated heritability explained by all identified variants in GWAS published in 2012^8^. Another noteworthy result was that about 15% of the heritability of schizophrenia could be accounted for by four CNVs, although this value was smaller than the previously estimated heritability (23%) explained by all identified variants in GWAS published in 2012^9^. With regard to schizophrenia, it turned out that the h_p_^2^ of a CNV that was detected only in patients (OR = +∞) is large. The results in the analyses suggest that a large part of missing heritability of common diseases could be accounted for by a kind of CNVs. 15q13.3 microdeletions has been reported to be associated not only with schizophrenia but also with idiopathic generalized epilepsy (IGE)^2^,^10^. Although the accurate data of prevalence of IGE that contains several types of epilepsies could not be obtained, h_p_^2^ of IGE was estimated to be 0.13–0.15 (not shown in Table 2). CNVs have been suspected to be involved in the pathophysiology of neuropsychiatric conditions^11^ The results of trial estimation of the h_p_^2^ of a polymorphism suggest that CNVs might be the major genetic cause of neuropsychiatric disorders.

### Comparison of the required number of polymorphisms to explain a heritability

Previous studies have estimated the heritability of sets of polymorphisms. Pawitan *et al.* showed how many variants were needed to explain a heritability of 0.4 in 2009^12^. In order to confirm that the calculated results by using the method described in the present study are consistent with those generated using other approaches, the required numbers of genetic variants under the AD model to explain a heritability of 0.4, when the prevalence of a disease is 0.01, were estimated. In this estimation the additive effect of each h_p_^2^ was considered, in the other words, the “narrow sense” heritability was tried to be accounted for. The results by the method in the present study were shown comparing with those of Pawitan *et al.* in Table 3. The required number of genetic variants calculated using the median of the range of variants in a category was not different from their approximation for the same category except for the common variants of category 1.

## Discussion

The estimations of heritability of polymorphisms were mainly conducted for the SNPs that were found in GWAS^1^,^2^,^3^,^12^,^13^. It is thought that the heritability of common diseases is due to multiple genes of small effect size and that even qualitative disorders can be interpreted simply as being the extremes of quantitative dimensions, that is, by the quantitative genetic analysis^14^. Recent studies demonstrated the interaction effects and the collective effects of SNPs in quantitative genetic traits^15^,^16^,^17^. However, I discuss here the conventional quantitative analysis under the premise that there are simple additive effects of polymorphisms. In quantitative genetic analysis authors have assumed a latent susceptibility (or liability) that varies between individuals^12^. The liability can be due to genetic and environmental factors, and heritability is defined as the proportion of the variance in liability due to genetic factors. For calculation of liability that is contributed by a SNP, OR of allele frequency or OR of risk genotype for a SNP is the fundamental factor for estimating the penetrance in the analysis^12^,^13^. Therefore when a SNP was detected only in patients (OR = +∞), the calculation is theoretically impossible in the quantitative genetic analysis. After all the quantitative effect of genes with a small effect size is being handled in the analysis and the participation of gene with such a large effect size (OR = +∞) is not assumed. Wellcome Trust Case Control Consortium *et al.* published in 2010 the estimation of heritability of common CNVs, and they did not take into the consideration for the CNVs that were detected only in patients, either^4^. However, CNVs are sometimes detected only in the patients as shown in Table 2.

In this report a novel method to calculate heritability of a single polymorphism was shown. A trial to estimate the required numbers of genetic variants under the AD model to explain a heritability showed that the calculation results by using the method described in the present study are entirely consistent with those generated by a quantitative genetic analysis (Table 3). I did not introduce the penetrance in the calculation procedure but introduced the population attributable risk that would not be infinity when OR is +∞. By the method in the present report it was suggested that heritability of some CNVs are quite large when it was calculated under the AD model. The heredity form of CNVs is often unknown, and only an OR of allele frequency for a CNV is usually available. Although by the calculation of heritability of CNVs only under the AD model, it was suggested a large part of missing heritability could be accounted for by re-evaluating the CNVs which have been already found and by searching novel CNVs with large h_p_^2^. The results of this study also suggest that CNVs might be the major genetic cause of neuropsychiatric disorders. In conclusion, CNVs were turned out to play important roles in familial aggregation of common diseases.

## Methods

### Calculation of genotype probabilities for a sibling

For the purpose of calculation of genotype probabilities for a sibling, an application of Beye’s method is necessary. An example of the calculation of genotype probabilities by Beye’s method for the father of the proband is shown in Table 4. As a result the posterior probability equals to the frequency of another allele (A or a) of the transmitted one (A) in the general population.

Then the genotype probabilities for a sibling are calculated. The calculation procedure of the genotype probabilities for a sibling was shown in Table 5. In Table 5, P1 and P2 are the posterior probabilities of genotypes of father and mother, respectively, and P3 is a conditioned probability of genotype of sibling. A joint probability is the product of F, P1, P2, and P3. The summation of joint probabilities for each genotype was shown in Table 1.

### Calculation of genotype probabilities for an offspring

For calculation of genotype probabilities for an offspring the Beye’s method is not needed. The calculation procedure of the genotype probabilities for an offspring was shown in Table 6. The summation of joint probabilities for each genotype was shown in Table 1.

### An example of calculation of heritability of a polymorphism

As an example of a common disease, let us choose schizophrenia. The prevalence, **P**, of schizophrenia is reported as 0.01. Here, CNV (16p11.2 dup) is chosen as an example of a polymorphism^18^. The frequency of a risk allele in patients, u, is 0.0039 and the frequency of a risk allele in asymptomatic individuals, x, is 0. Therefore p is calculated as 0.000039 using Equation [1]. By the way, **P** of schizophrenia (1%) is more than +2.32635SD of a general population. The mean distance from the median in the normal distribution is calculated as +2.6652SD for the patients. The incidence rate under the autosomal dominant model of the disease in a first-degree relative, if we consider only for the effect of the CNV, is represented by Formula [7]:


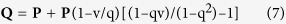


The incidence rate of schizophrenia is calculated as following:


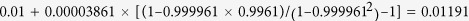


This value can be used as a recurrence risk of the disease in first-degree relatives and is more than +2.25998SD. Then heritability (h_p_^2^) of CNV (16p11.2 dup) is calculated by Falconer’s liability threshold model, and the result is as following^6^:


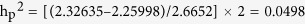


## Additional Information

**How to cite this article**: Nagao, Y. Copy number variations play important roles in heredity of common diseases: a novel method to calculate heritability of a polymorphism. *Sci. Rep.* **5**, 17156; doi: 10.1038/srep17156 (2015).

## Figures and Tables

**Table 1:** Probability of each genotype of a sibling and an offspring.

Genotype	Probability
	Sibling
AA	{α(1 + p)^2^ + βp(1 + p) + γp^2^}/4
Aa	{α(1 + p)q + β(1 + pq) + γp(1 + q)}/2
aa	{αq^2^ +β(1 + q)q + γ(1 + q)^2^}/4
	**Offspring**
AA	αp + βp/2
Aa	αq + β/2 + γp
aa	βq/2 + γq

A and a represent dominant and recessive allele, respectively. α, β and γ represent the genotype frequency of AA, Aa and aa of the proband, respectively.

**Table 2:** Results of a trial to calculate h_p_^2^ of CNVs and SNPs using published data.

Disease	CNV (locus or description) or SNP (rs number or description)	Population or source	OR	P	p	h_p_^2^
Autism	• CNV* (16p11.2 del)^3^,^19^	Americans	100	0.006	0.00016	0.0679
	• CNV (16p11.2 dup)^3^,^19^	Americans	16	0.006	0.00034	0.0077
	• SNP (rs4307059)^20^	Americans	1.19	0.0067	0.61	0.00049
	• SNP (rs10513025)^21^	Several sources	0.55	0.015	0.063	0.0036
Depression	• CNV (3q13.33 dup)^22^	Hungarians	5.27	0.085	0.013	0.0327
	• SNP (rs2251219)^23^	(Meta-analysis)	0.87	0.20	0.40	0.0014
Schizophrenia	• CNV (16p11.2 dup)^18^	Several sources	∞	0.01	0.000039	0.0498
	• CNV (22q11.2 del)^18^	Several sources	∞	0.01	0.000035	0.0377
	• CNV (NRXN1 del)^18^	Several sources	∞	0.01	0.000016	0.0213
	• CNV (AS/PWS dup)^18^	Several sources	∞	0.01	0.000012	0.0161
	• CNV (15q11.3 del)^10^	Several sources	8.27	0.01	0.00021	0.0021
	• CNV (1p21.1 del)^18^	Several sources	11.03	0.01	0.000175	0.0019
	• SNP (ADAMTSL3)^24^	(HapMap)	0.68	0.01	0.29	0.0046
	• SNP (rs17504622)^25^	Swedish	1.24	0.01	0.05	0.00035
Obsessive-compulsive disorder	• CNV (13q14.2 del)^26^	Swiss	6.23	0.023	0.010	0.0405
	• SNP (rs6311, located on HTR2A promoter)^26^	Swiss	1.69	0.023	0.44	0.0087
Sporadic ALS	• CNV (10q15.3 dup)^27^	Japanese	5.49	0.0001	0.101	0.0625
	• SNP (rs10260404)^28^	Dutch	1.30	0.0001	0.27	0.00050
Type 2 diabetes mellitus	• CNV (4p16.3 del)^29^	Japanese	14.8	0.10	0.022	0.1594
	• SNP (missence variant of HNF1A gene)^30^	Mexicans and US Latinos	5.48	0.14	0.0060	0.0146

Odds ratio (OR), risk allele frequency (p), and prevalence of disease (**P**) of each polymorphism are cited from the literatures^3^,^10^,^18^,^19^,^20^,^21^,^22^,^23^,^24^,^25^,^26^,^27^,^28^,^29^,^30^. **P** of schizophrenia is cited from a review^31^.

*de novo CNV.

**Table 3:**
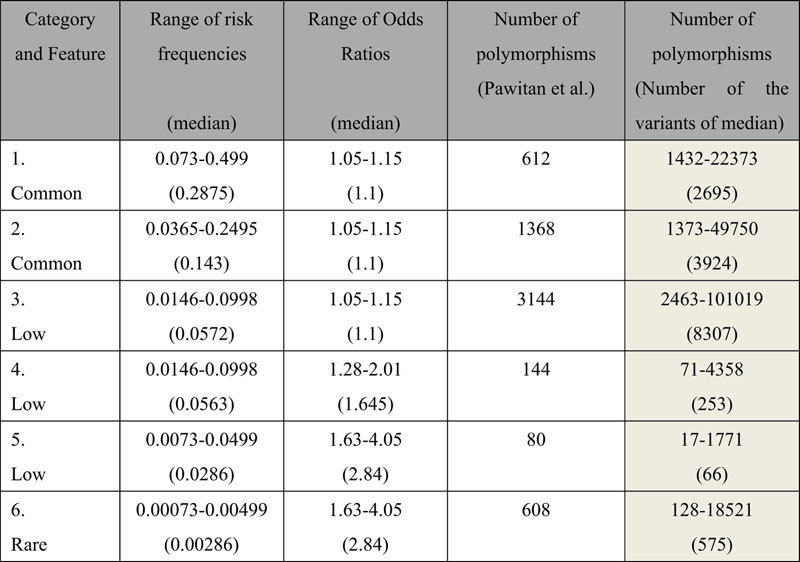
Various categories of variants and the number of variants to explain heritability of 0.4.

Categories of variants are based on the classification by Pawitan *et al.*^12^. The background of the calculated results by the method in this study is applied gray.

**Table 4:** An example of the calculation of genotype probabilities by Beye’s method when the genotype of the proband is AA.

	AA	Aa	aa
Prior Probability	pp	2pq	qq
Conditioned Probability (of transmitting allele A)	1	0.5	0
Joint Probability	pp	pq	0
Posterior Probability	pp/(pp + pq) = p	pq/(pp + pq) = q	

As a result the posterior probability equals to the frequency of another allele (A or a) of the transmitted one (A) in the general population.

**Table 5:** The calculation procedure of the genotype probabilities for a sibling.

Proband	F	Father	P1	Mother	P2	Sibling	P3	Joint Probability
AA	α	AA	p	AA	p	AA	1	αpp
				Aa	q	AA	0.5	0.5αpq
						Aa	0.5	0.5αpq
		Aa	q	AA	p	AA	0.5	0.5αpq
						Aa	0.5	0.5αpq
				Aa	q	AA	0.25	0.25αqq
						Aa	0.5	0.5αqq
						aa	0.25	0.25αqq
Aa	β	AA	pp	Aa	p	AA	0.5	0.5βppp
						Aa	0.5	0.5βppp
				aa	q	Aa	1	βppq
		Aa*	pq	Aa	p	AA	0.25	0.25βppq
						Aa	0.5	0.5βppq
						aa	0.25	0.25βppq
				aa	q	Aa	0.5	0.5βpqq
						aa	0.5	0.5βpqq
		Aa^†^	pq	AA	p	AA	0.5	0.5βppq
						Aa	0.5	0.5βppq
				Aa	q	AA	0.25	0.25βpqq
						Aa	0.5	0.5βpqq
						aa	0.25	0.25βpqq
		aa	qq	AA	p	Aa	1	βpqq
				Aa	q	Aa	0.5	0.5βqqq
						aa	0.5	0.5βqqq
aa	γ	Aa	p	Aa	p	AA	0.25	0.25γpp
						Aa	0.5	0.5γpp
						aa	0.25	0.25γpp
				aa	q	Aa	0.5	0.5γpq
						aa	0.5	0.5γpq
		aa	q	Aa	p	Aa	0.5	0.5γpq
						aa	0.5	0.5γpq
				aa	q	aa	1	γqq

^*^Allele A is derived from the father.

^†^Allele a is derived from the father.

F; a frequency of genotype of the proband.

P1; a posterior probability of genotype of father.

P2; a posterior probability of genotype of mother.

P3; a conditioned probability of genotype of sibling.

**Table 6:** The calculation procedure of the genotype probabilities for an offspring.

Proband	F	Spouse	P1	Offspring	P2	Joint Probability
AA	α	AA	pp	AA	1	αpp
		Aa	2pq	AA	0.5	αpq
				Aa	0.5	αpq
		aa	qq	Aa	1	αqq
Aa	β	AA	pp	AA	0.5	0.5βpp
				Aa	0.5	0.5βpp
		Aa	2pq	AA	0.25	0.5βpq
				Aa	0.5	βpq
				aa	0.25	0.5βpq
		aa	qq	Aa	0.5	0.5βqq
				aa	0.5	0.5βqq
aa	γ	AA	pp	Aa	1	γpp
		Aa	2pq	Aa	0.5	γpq
				aa	0.5	γpq
		aa	qq	aa	1	γqq

F; a frequency of genotype of the proband.

P1; a probability of genotype of spouse.

P2; a conditioned probability of genotype of offspring.
